# The Impacts of Sun Exposure on Worker Physiology and Cognition: Multi-Country Evidence and Interventions

**DOI:** 10.3390/ijerph18147698

**Published:** 2021-07-20

**Authors:** Leonidas G. Ioannou, Lydia Tsoutsoubi, Konstantinos Mantzios, Giorgos Gkikas, Jacob F. Piil, Petros C. Dinas, Sean R. Notley, Glen P. Kenny, Lars Nybo, Andreas D. Flouris

**Affiliations:** 1 FAME Laboratory, Department of Physical Education and Sport Science, University of Thessaly, 42100 Trikala, Greece; ioannouLG@gmail.com (L.G.I.); lydiatsoutsoubi@gmail.com (L.T.); konstantinosmantzios@gmail.com (K.M.); ggkikas@uth.gr (G.G.); petros.cd@gmail.com (P.C.D.); 2 Department of Nutrition, Exercise and Sports, August Krogh Building, University of Copenhagen, 2100 Copenhagen, Denmark; jpp@nexs.ku.dk (J.F.P.); nybo@nexs.ku.dk (L.N.); 3 Human and Environmental Physiology Research Unit, Faculty of Health Sciences, University of Ottawa, Ottawa, ON K1N 6N5, Canada; snotley@uottawa.ca (S.R.N.); gkenny@uottawa.ca (G.P.K.); 4 Clinical Epidemiology Program, Ottawa Hospital Research Institute, Ottawa, ON K1H 8L6, Canada

**Keywords:** solar radiation, heat, occupational, labor, performance, core temperature, skin temperature, heart rate, skin blood flow, sweat rate

## Abstract

Background: A set of four case-control (*n* = 109), randomized-controlled (*n* = 7), cross-sectional (*n* = 78), and intervention (*n* = 47) studies was conducted across three countries to investigate the effects of sun exposure on worker physiology and cognition. Methods: Physiological, subjective, and cognitive performance data were collected from people working in ambient conditions characterized by the same thermal stress but different solar radiation levels. Results: People working under the sun were more likely to experience dizziness, weakness, and other symptoms of heat strain. These clinical impacts of sun exposure were not accompanied by changes in core body temperature but, instead, were linked with changes in skin temperature. Other physiological responses (heart rate, skin blood flow, and sweat rate) were also increased during sun exposure, while attention and vigilance were reduced by 45% and 67%, respectively, compared to exposure to a similar thermal stress without sunlight. Light-colored clothes reduced workers’ skin temperature by 12–13% compared to darker-colored clothes. Conclusions: Working under the sun worsens the physiological heat strain experienced and compromises cognitive function, even when the level of heat stress is thought to be the same as being in the shade. Wearing light-colored clothes can limit the physiological heat strain experienced by the body.

## 1. Introduction

The long-term health effects of sun exposure have been extensively studied, particularly in relation to skin cancer and cataract, but there is little evidence-based knowledge on the acute impacts of sun exposure. For instance, a laboratory study showed marked negative effects of solar radiation on human cognitive performance [1], but we do not know if the heat from the sun can affect the physiology and cognition of people working outdoors [2,3]. The World Health Organization and the International Labor Organization are developing joint methodologies for estimating the associated work-related burden of disease and injury. However, practical and economically feasible protection strategies for people working outdoors have not been investigated at the size and quality needed to draw robust conclusions and recommendations [4].

Agriculture and construction include the vast majority of employees exposed to the sun [2,3] due to the size of these industries, the requirement to work outdoors, and the lack of cost-effective shading solutions for these occupational settings. The associated societal and economic impacts are widespread. The agricultural sector alone, employing one-third of the world’s labor force [5], is projected to account for 60% of global working hours lost to heat stress in 10 years from now, whereas construction is expected to account for 19% of such loss [3]. These estimates translate to 63 million full-time jobs lost across the globe, with an associated monetary loss of US$ 1.9 trillion in purchasing power parity terms [3,6]. While these figures are astounding, they almost certainly underestimate the phenomena that will occur within the next 10 years for two reasons. First, these projections assume that the increase in global mean temperature at the end of the century will not exceed 1.5 °C (2.7 °F) of pre-industrial levels [3,7]. Unfortunately, it is now clear that global temperature is already 1.0 °C (1.8 °F) above pre-industrial levels, and it is likely to reach 1.5 °C between 2030 and 2052 if it continues to increase at the current rate [8]. This climate change is also expected to increase people’s exposure to the sun, particularly for those who work outdoors [9]. Second, they assume that work in agriculture and construction is carried out in the shade. However, recent data confirmed that these employees perform the vast majority of their work outdoors and are directly affected by sun exposure, leading to increased heat strain and impaired capacity for manual labor [10,11,12,13].

Based on these important knowledge gaps for addressing sun exposure and heat stress emergencies, the goals of this article are to provide health advisors and medical readers with evidence-based information on (i) the effects of sun exposure on worker physiology and cognition and (ii) a practical and economically feasible protection strategy for the most vulnerable individuals.

## 2. Materials and Methods

The goals of this paper are achieved by presenting relevant findings from a series of studies carried out in different industries and countries that include two large field trials, one randomized controlled trial in laboratory settings, and two field interventions. A set of four interconnected studies was conducted as follows:

### 2.1. Aims of the Studies

- Study 1: The effects of solar radiation on the psychophysical stress experienced by workers who perform manual labor in construction and agriculture. The aim of this study was to investigate the effects of solar radiation on human psychophysical stress during actual work shifts in the heat.
- Study 2: The effects of solar radiation on physiological responses and cognitive function at rest and during physical work. The aim of this study was to perform a controlled, laboratory-based evaluation of the effects of solar radiation on human physiological responses and cognitive performance at rest, during physical work, and post-work recovery by comparing indoor (i.e., without solar radiation) and outdoor (i.e., typical mid-day solar radiation) environments characterized by the same thermal stress.
- Study 3: Identifying factors increasing the adverse effects of sun exposure experienced by agriculture and construction workers. The aim of this study was to investigate possible factors exacerbating the effects of sunlight-induced thermal strain by investigating workers’ behavioral habits during actual work shifts in occupational settings.
- Study 4: Interventions to mitigate the sunlight-induced heat strain experienced by people who work in agriculture and construction. The aim of this study was to test interventions to mitigate the sunlight-induced heat strain experienced by workers who work in agriculture and construction.

### 2.2. Experimental Protocol

- Study 1: Detailed information on Study 1, including information about the physiological data we collected as well as supplemental tables and figures, is presented in Appendix A. This case-control study involved monitoring 109 experienced and heat acclimatized agriculture and construction workers during four to five consecutive full 11-h work shifts. Physiological, subjective, labor, and environmental data were collected throughout the study. Work hours were characterized by the same thermal stress, but different solar radiation levels were isolated to examine if solar radiation levels can independently modify the physiological heat strain experienced by workers.
- Study 2: Detailed information on Study 2, including information about the physiological data we collected as well as supplemental tables and figures, is reported in Appendix B. This single-blinded randomized controlled trial involved tracking seven participants during exposure to four different environmental conditions (two hot (30 °C wet-bulb globe temperature (WBGT) with and without solar radiation) and two temperate (20 °C WBGT with and without solar radiation) ambient conditions) allocated in random order. Physiological and subjective data were collected throughout the experiments. This study was conducted to confirm and complement the findings of Study 1 by delineating the physiological and cognitive impacts of sun exposure on people who perform manual work in environments characterized by the same thermal stress but different solar radiation levels.
- Study 3: Detailed information on Study 3 is overviewed in Appendix C. This cross-sectional study involved monitoring 78 agriculture workers from seven countries over a period of three months to examine the color of their clothing, a key factor mediating heat exchange, during actual work shifts performed outdoors.
- Study 4: Detailed information on Study 4 is outlined in Appendix D. This intervention study was conducted to investigate if changes in the color of workers’ clothing can modify the physiological heat strain experienced by people who work under the sun. The study involved monitoring 47 outdoor workers during two work shifts (“business as usual” and “white clothing” scenarios) characterized by the same thermal stress and solar radiation levels. Physiological, labor, and environmental data were collected to investigate if white clothes can reduce the physiological heat strain experienced by people who work under the sun.

## 3. Results

**Figure 1:**
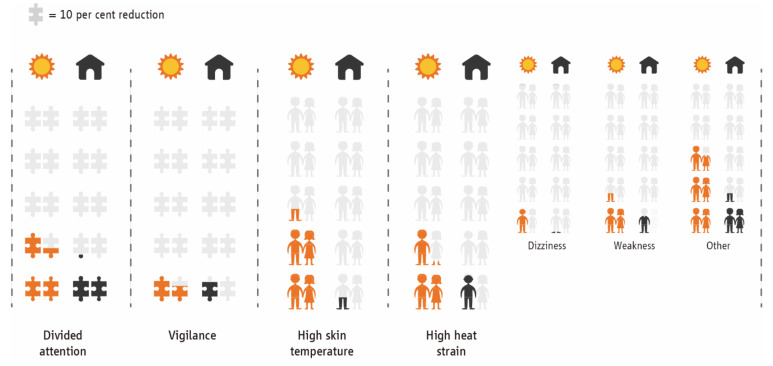
Cognitive and health impacts while working under the sun (left column) and in the shade (right column) in a hot environment (30 °C WBGT). Each full-colored puzzle piece indicates a 10% reduction in cognitive performance (divided attention and vigilance). Each full-colored body figure indicates one-in-ten workers experiencing mean skin temperature higher than 36 °C (the threshold for progressive symptoms of heat strain [14,15]) or danger-level heat strain, including dizziness, weakness, or other symptoms.

Sun exposure increases the physiological heat strain experienced by workers and compromises their cognitive function, even when the level of heat stress is thought to be the same as being in the shade. An overview of our findings is presented in Figure 1, while detailed information is provided under the subheading dedicated to each study.

### 3.1. Results of Study 1: Effects of Solar Radiation on the Psychophysical Stress Experienced by Workers who Perform Manual Labor in Construction and Agriculture

**Figure 2:**
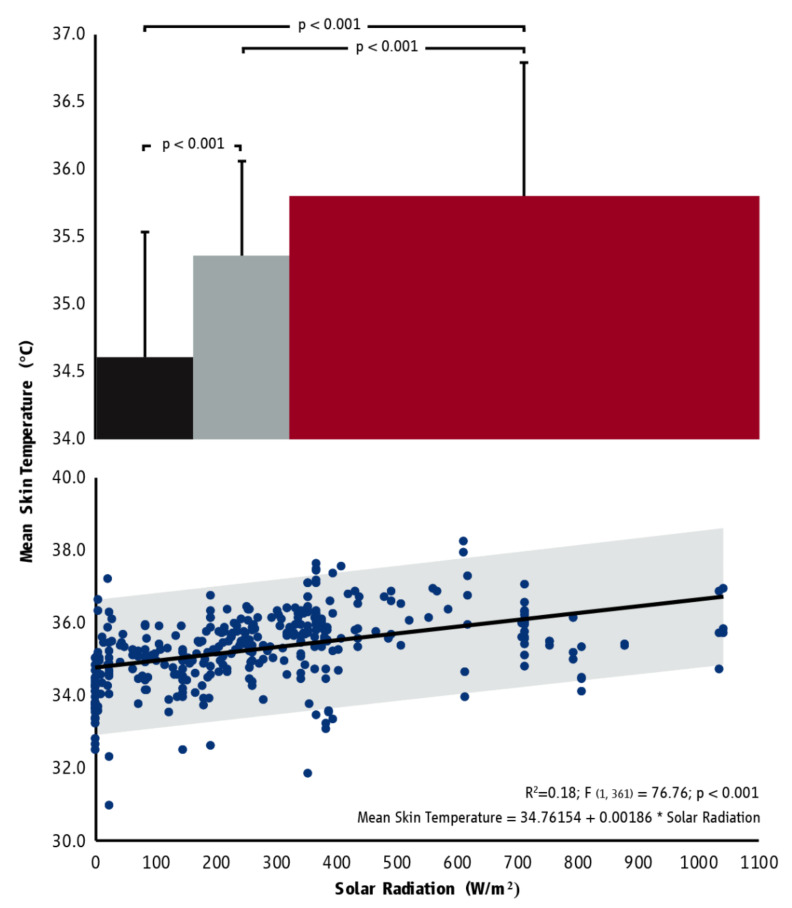
The average (±SD) mean skin temperature in indoor (black), mixed (grey), and outdoor (red) environments characterized by the same thermal stress (30 °C WBGT) but different solar radiation levels (top graph) as well as the association between hourly mean skin temperature and solar radiation (bottom graph). The bar width in the top graph indicates the range of solar radiation of each category corresponding to the horizontal axis, while horizontal brackets indicate statistically significant differences. Shading in the bottom graph corresponds to the 95% prediction interval.

**Figure A1:**
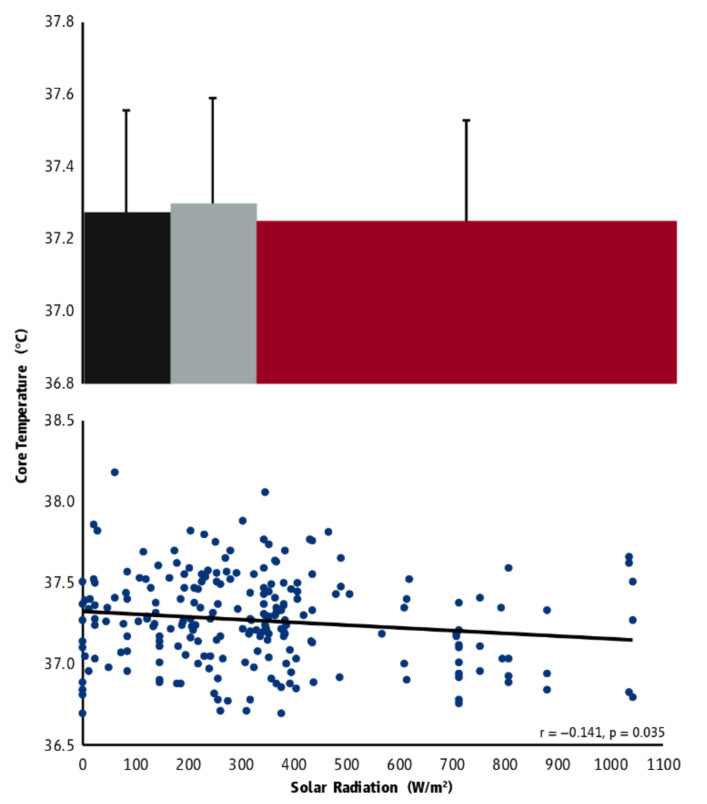
The average (±SD) core temperature in indoor (black), mixed (grey), and outdoor (red) environments characterized by the same thermal stress (top graph) as well as the association between hourly core temperature and solar radiation (bottom graph). Bar width in the top graph indicates the range of solar radiation of each category corresponding to the horizontal axis.

**Figure A2:**
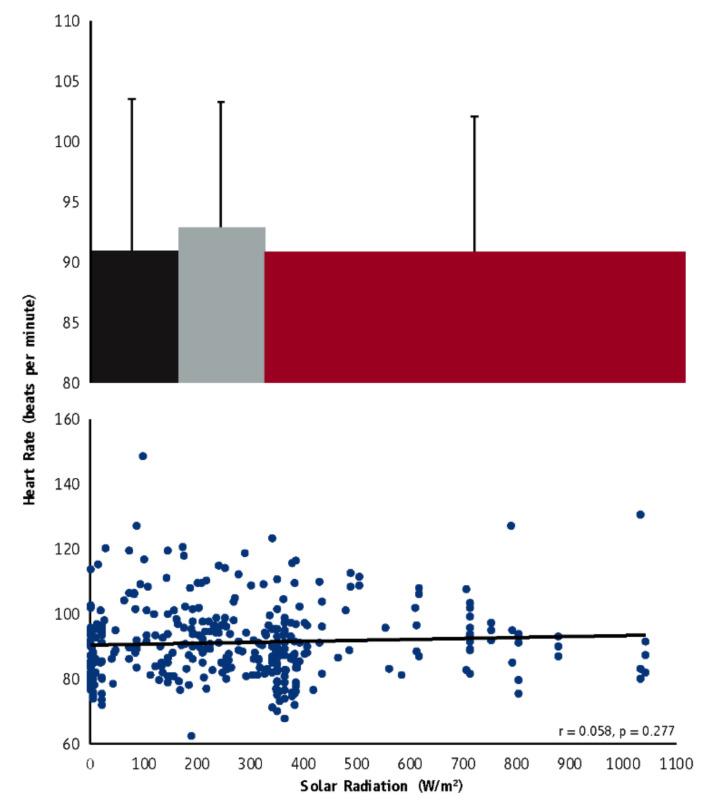
The average (±SD) heart rate in indoor (black), mixed (grey), and outdoor (red) environments characterized by the same thermal stress (top graph) as well as the association between hourly heart rate and solar radiation (bottom graph). Bar width in the top graph indicates the range of solar radiation of each category corresponding to the horizontal axis.

**Figure A3:**
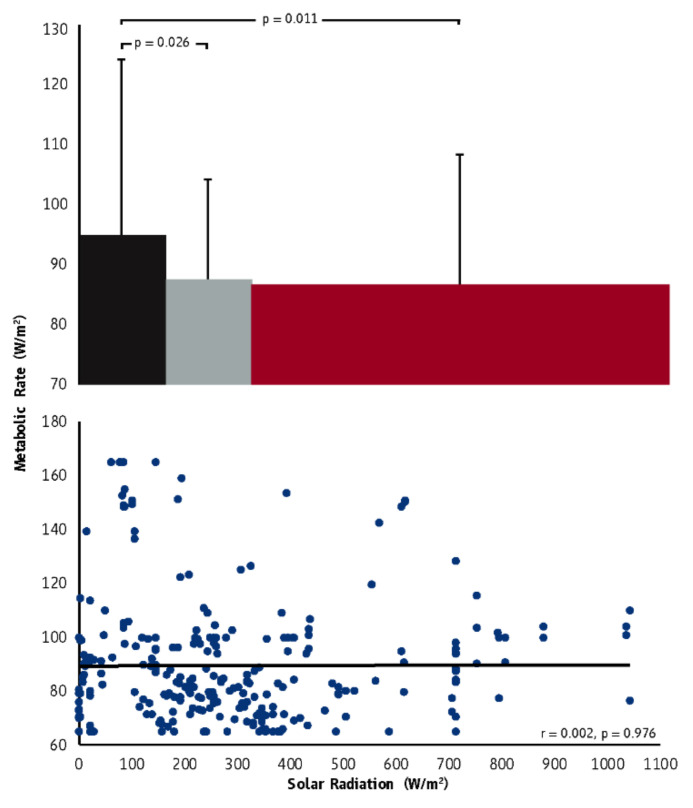
The average (±SD) metabolic rate in indoor (black), mixed (grey), and outdoor (red) environments characterized by the same thermal stress (top graph) as well as the association between hourly metabolic rate and solar radiation (bottom graph). Bar width in the top graph indicates the range of solar radiation of each category corresponding to the horizontal axis, while horizontal brackets indicate statistically significant differences.

A group of 109 workers (Table A1) participated in the study. During the study, 396 h (collected from 98 workers) were identified as having equal thermal stress (30 °C WBGT) but different solar radiation levels (ranging from 0 W/m^2^ to 1043 W/m^2^). Of the 396 h, 108 (27.3%) were successive hours. Solar radiation levels were positively associated with mean skin temperature (T_sk_) (r = 0.419, *p* < 0.001; Figure 2). This association was characterized by an increase of ~0.2 °C for every 100 W/m^2^ increase in solar radiation levels (Figure 2). On the other hand, a negligible negative association was found between solar radiation levels and core body temperature (T_core_) as estimated by gastrointestinal temperature (r = −0.141, *p* = 0.035; Figure A1). Additionally, no associations were identified between solar radiation levels and heart rate (Figure A2) or metabolic rate (Figure A3). Perceived thermal radiation was significantly related to specific items of the Heat Strain Score Index [16] evaluating subjective psychophysical parameters (Table A2). Moreover, significant differences in T_sk_ (Figure 2) and labor intensity (Figure A3) were identified between indoor (0 to 160 W/m^2^), mixed (161–320 W/m^2^), and outdoor (>320 W/m^2^) environments (F_(2, 360)_ = 57.791, *p* < 0.001).

Although we tested the same workers working in environments characterized by the same thermal stress (30 °C WBGT), having a Heat Strain Score Index greater than 18 (indicating dangerously high risk of experiencing heat strain) was 3.61 times more likely when working outdoors as compared to indoors (Figure 1). Even more so, having a T_sk_ above 36 °C (indicating dangerously high risk of experiencing heat strain [14,15]) was 10.16 times more likely when working outdoors as compared to indoors (Figure 1). Similarly, the risk for experiencing dizziness, weakness, and other heat strain symptoms (i.e., mild headache, muscle pain, the appearance of red acne, and reduced mental concentration) are 4.44, 3.17, and 2.40 times higher, respectively, when working outdoors compared to indoors (Table A3).

### 3.2. Results of Study 2: Effects of Solar Radiation on Physiological Responses and Cognitive Function at Rest and during Physical Work

**Figure 3:**
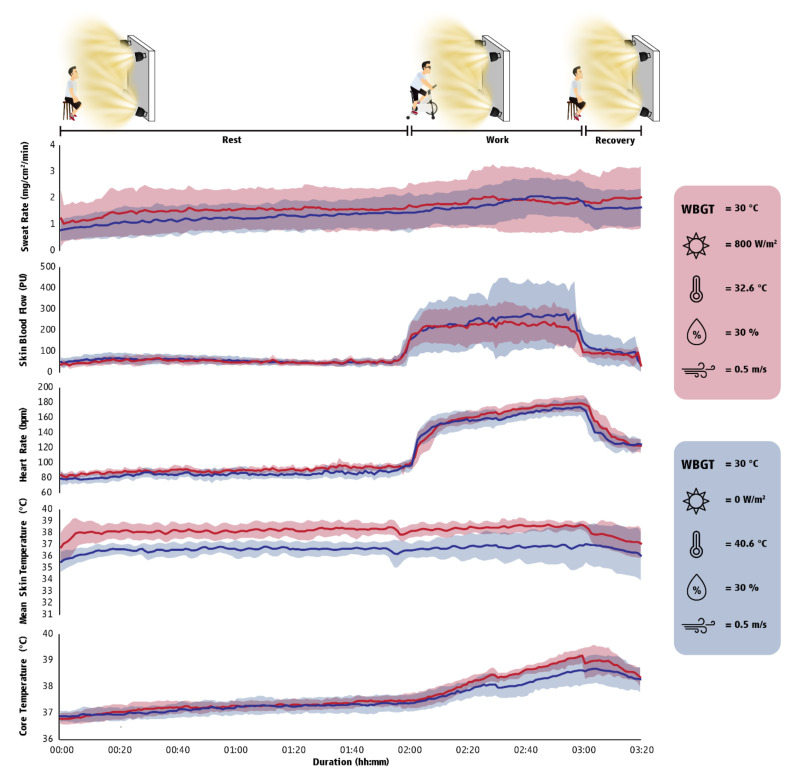
Physiological responses (mean ± SD) during exposure in a hot environment (30 °C WBGT). The first two hours (00:00 to 02:00) illustrate the fluctuation of physiological responses in resting conditions, the third hour (02:00–03:00) illustrate physiological responses during exercise/work, while the final 20 min (03:00–03:20) show the responses during recovery time. Red indicates a hot outdoor environment, while blue indicates a hot indoor environment. Sweat rate corresponds to the average sweat rate from the forehead, arm (bicep), and thigh (quadricep) as measured using the ventilated capsule technique, expressed in milligrams per centimeter square per minute. Skin blood flow as measured by laser-Doppler flowmetry corresponds to the average skin blood flow from the forearm (brachioradialis) and leg (gastrocnemius), expressed in perfusion units. Heart rate is expressed in beats per minute. Mean skin temperature estimated from arm, chest, thigh, and leg skin temperatures, expressed in degrees Celsius. Core temperature corresponds to gastrointestinal temperature, expressed in degrees Celsius. Effect sizes for all comparisons between outdoor and indoor environments can be found in Table A5.

**Figure 4:**
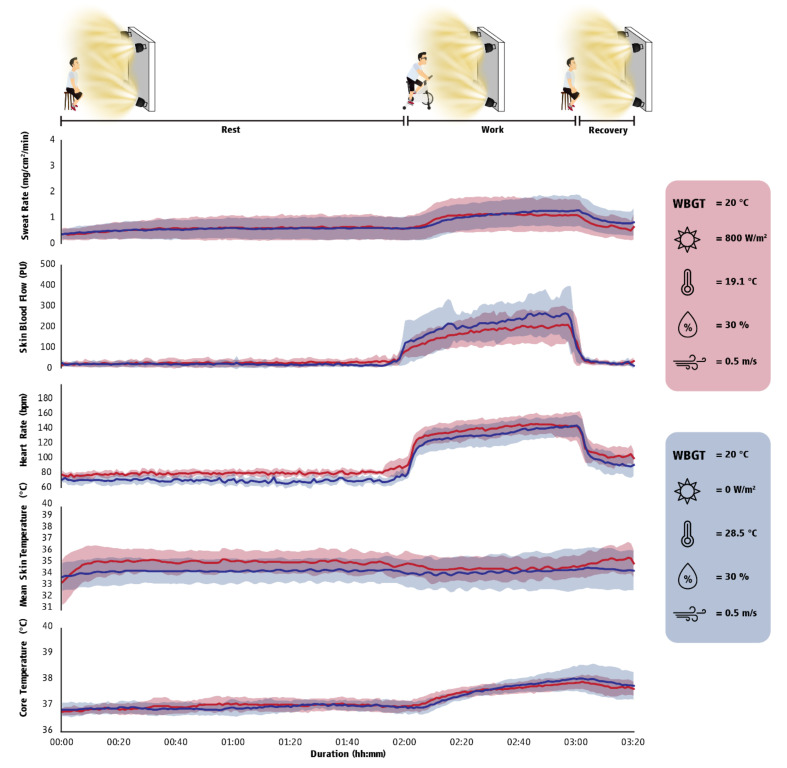
Physiological responses (mean ± SD) during exposure in a temperate environment (20 °C WBGT). The first two hours (00:00 to 02:00) illustrate the fluctuation of physiological responses in resting conditions, the third hour (02:00–03:00) illustrate physiological responses during exercise/work, while the final 20 min (03:00–03:20) show the responses during recovery time. Red indicates a thermoneutral outdoor environment, while blue indicates a thermoneutral indoor environment. Sweat rate corresponds to the average sweat rate from the forehead, arm (bicep), and thigh (quadricep) as measured using the ventilated capsule technique, expressed in milligrams per centimeter square per minute. Skin blood flow as measured by laser-Doppler flowmetry corresponds to the average skin blood flow from the forearm (brachioradialis) and leg (gastrocnemius), expressed in perfusion units. Heart rate is expressed in beats per minute. Mean skin temperature estimated from the arm, chest, thigh, and leg skin temperatures, expressed in degrees Celsius. Core temperature corresponds to gastrointestinal temperature, expressed in degrees Celsius. Effect sizes for all comparisons between outdoor and indoor environments can be found in Table A5.

The anthropometric characteristics of the seven volunteers that participated in the study were as follows: age: 22.7 ± 3.2 years; body stature: 177.6 ± 6.1 cm; body mass: 74.3 ± 8.9 kg; body fat: 20.1 ± 6.7%; and lean mass: 56.7 ± 5.1%. Although participants were exposed to environments characterized by equal thermal stress, we identified that exposure to solar radiation had an incremental effect on the mean skin temperature (Figure 3 and Figure 4) of the participants impairing their cognitive performance (Table A5). Furthermore, we found that cognitive performance was positively (i.e., participants made more mistakes when their T_sk_ and T_core_ were increased) related to T_sk_ and T_core_ (Table A6).

### 3.3. Results of Study 3: Identifying Factors Increasing the Adverse Effects of Sun Exposure Experienced by Agriculture and Construction Workers

A group of 78 agriculture workers (age: 42.4 ± 13.0 years; height: 166.1 ± 11.5 cm; and weight: 70.5 ± 19.6 kg) was monitored for a total of 112 work shifts over a period of three months. We identified that more than two-thirds (68.8%) of the monitored workers wore dark-colored clothes during work under the sun. This was recognized as an important factor increasing the thermal strain experienced by workers. It is important to note that this is against prevailing recommendations and may reflect the lack of knowledge of workers and employers regarding occupational health and safety.

### 3.4. Results of Study 4: Interventions to Mitigate the Sunlight-Induced Heat Strain Experienced by Workers Who Work in Agriculture and Construction

A group of six agriculture (two females) and 41 construction (all males) workers participated in the study (Table A7). The two scenarios (“business as usual” (BAU) and “white clothing” (CLO)) were characterized by similar thermal stress (agriculture: ~23 ± 4 °C WBGT; and construction: ~28 ± 4 °C WBGT) and solar radiation levels (agriculture: ~920 ± 300 W/m^2^; and construction: ~200 ± 150 W/m^2^). Moreover, task analysis identified that workers performed similar manual work (agriculture (BAU: 194 ± 54 W/m^2^ vs. CLO: 189 ± 53 W/m^2^) and construction (BAU: 96 ± 17 W/m^2^ vs. CLO: 86 ± 17 W/m^2^)) during both scenarios. We identified no significant differences in the T_core_ of workers between the tested scenarios (agriculture (BAU: 37.3 ± 0.3 °C vs. CLO: 37.2 ± 0.3 °C) and construction: (BAU: 37.3 ± 0.2 °C vs. CLO: 37.4 ± 0.2 °C)). Importantly, although workers were exposed to environments characterized by the same thermal stress and doing the same labor, workers donning white uniforms experienced a reduced level of physiological heat strain (i.e., small reductions in mean skin temperature and some minor improvement in thermal sensation; Table A8). Furthermore, the average change in T_sk_ (from resting conditions) was 13% and 12% lower during the white clothing scenario compared to the business-as-usual scenario in agriculture and construction, respectively.

## 4. Discussion

To investigate the physiological and health impacts of sun exposure on workers performing jobs/duties outdoors, a series of four separate but interconnected studies were conducted across different industrial sectors and countries. A large-scale occupational field trial was conducted in Qatar involving 109 construction and agricultural workers. From the collected data, we compared individuals working in the shade versus those working under the sun. We also isolated 396 work hours characterized by the same high level of heat stress (30 °C WBGT; typical for a Northern hemisphere heatwave) but very different levels of sun exposure: 33% were in the shade, and 67% were under the sun. We found that people working under the sun were four times more likely to experience dizziness (i.e., vertigo, presyncope, disequilibrium, or other non-specific feelings), three times more likely to report weakness, and twice more likely to suffer other symptoms of heat strain (i.e., mild headache, muscle pain, the appearance of red acne, and reduced mental concentration), compared to performing the same work in the shade under the same level of heat stress. These clinical impacts of sun exposure were not accompanied by changes in core body temperature but, instead, were linked with changes in skin temperature, which was 10 times more likely to be at levels indicative of heat strain (>36 °C) when working under the sun.

Skin temperature is an important parameter linked with both physiological and psychophysical stress [14,15,17]. An increasing number of reports over the last decade have highlighted the importance of high skin temperature as an early indicator of hyperthermia and heat injury, as well as for regulating the intensity of work and exercise [13,17,18,19,20,21,22]. Recent data from the European Commission project HEAT-SHIELD [23] show that higher skin temperature is linked with reduced capacity to perform manual labor, leading to significant economic losses [13,24]. Overall, the findings from the first study show that working under the sun increases skin temperature and the risk for experiencing clinical symptoms of heat strain, albeit without markedly altering physiological heat strain as defined by changes in core temperature and heart rate, even in cases where the level of environmental heat stress is considered to be the same as working in the shade. This is probably related to the well-described effect of self-pacing that is known to act proactively to avoid an increase in workers’ core body temperature [13,25]. However, self-pacing may not be appropriate when jobs or tasks are time-sensitive, involve productivity incentives, and/or involve workers who are not well trained in their job [25,26].

To delve deeper into the physiological and cognitive impacts of sun exposure, as well as to better position the findings of our field experiments, we conducted in Greece a randomized controlled trial wherein seven healthy individuals were monitored during rest, moderate-intensity physical work, and post-work recovery inside a climate-controlled chamber. We compared values when participants were under the sun versus in the shade in temperate (20 °C WBGT) and hot (30 °C WBGT) ambient conditions. This study confirmed that sun exposure could elevate skin temperature without affecting core body temperature. Other physiological responses (heart rate, skin blood flow, and sweat rate) were also increased during sun exposure but to a smaller degree. More importantly, sun exposure reduced cognitive performance in both temperate and hot conditions, leading to a 45% reduction in divided attention (e.g., auditory and visual stimuli in parallel) and a 67% reduction in vigilance tasks (see Appendix B for measurement details). Literature suggests that environmental heat stress increases the risk of occupational injuries by promoting fatigue, reduced psychomotor performance, loss of concentration, and reduced alertness [27]. In total, the findings from the second study confirmed that sun exposure generates symptoms of heat strain, such as dizziness and weakness, and it also undermines cognitive function in both temperate and hot conditions, even when the level of heat stress is thought to be the same as being in the shade.

A practical and cost-effective strategy to limit the impact of sun exposure is to change the color of the clothing worn. Wearing white or light-colored clothes increases the reflection of heat from the sun [28,29,30,31,32] and can limit the heat strain (e.g., skin temperature, heart rate, and sweat rate) that the human body experiences during physical work in a hot environment [29,31]. Therefore, several heat stress guidelines recommend wearing light-colored clothes during work or exercise under the sun [30,33,34,35,36].

To assess attitudes of outdoor workers in relation to the choice of color of their clothing, we monitored 78 workers originating from seven countries (Bangladesh, Cyprus, Egypt, India, Philippines, Romania, and Vietnam) during 112 full work shifts performed outdoors in Cyprus during summer and autumn. Overall, the findings from the third study showed that 68.8% of the studied outdoor workers wore dark-colored clothes. This is against prevailing recommendations and reflects the lack of education and training on heat-related aspects of occupational health and safety for both workers and employers. Dark-colored clothes have high absorbance of radiative heat, and it is likely that workers would benefit from changing to light-colored work uniforms to minimize the adverse effects of sun exposure.

In an occupational field intervention study performed in Cyprus, we tested whether white clothes can be used as a practical and economically feasible strategy to limit the impacts of sun exposure in agricultural workers. We monitored six workers during two full work shifts characterized by temperate conditions. In the “business as usual” scenario, workers wore their preferred clothes. As expected, based on the results of the above-described observational study, these clothes were mostly black or dark-colored t-shirts and pants. In the “white clothes” scenario, workers were provided with white hats and t-shirts (all 100% cotton; total cost: US$ 8.80) and were instructed to wear light-colored pants of their own. The findings from the fourth study demonstrated that the change to white/light-colored clothing minimized the increase in mean skin temperature during work by 13% (corresponding to a reduction of 0.4 °C), despite that the participants worked at the same level of effort in the same outdoor conditions.

In a second occupational field intervention performed as part of our above-mentioned occupational field trial in Qatar [11], we tested the efficacy of wearing white coveralls for limiting the impacts of sun exposure in construction workers. We monitored 41 construction workers during two full work shifts characterized by moderate heat stress. In the “business as usual” scenario, workers wore their typical work coveralls (dark blue color in most cases) made of polycotton. In the “white coveralls” scenario, workers were provided with white coveralls (total cost: US$ 6.00) made of cotton or polycotton. This change to white coveralls minimized the increase in mean skin temperature during work by 12% (corresponding to a reduction of 0.2 °C), despite that the participants worked at the same level of effort in the same outdoor conditions. Taken together, these results support existing recommendations for wearing light-colored clothes during work or exercise under the sun [30,33,34,35,36] and further demonstrate their practicality and cost-effectiveness in occupational settings. While white work coveralls can be effective in reducing the physiological strain during work under the sun, the adoption of other heat mitigation strategies, such as hydration protocols, work–rest cycles for jobs that do not allow for self-pacing, ventilated garments, and mechanization of heavy work, can further reduce the physiological strain experienced by people working manually outdoors [25].

## 5. Conclusions

Medical staff are often asked whether occupational injuries are caused by exposure to the sun per se or by other parameters of heat stress such as high temperature or humidity. The field studies and laboratory-based clinical trials that we conducted in different parts of the world under both temperate and hot conditions show that working under the sun worsens the physiological heat strain experienced and compromises cognitive function, even when the level of heat stress is thought to be the same as being in the shade. To limit these detrimental impacts of sun exposure in cases where no other cost-effective shading solutions are available, medical staff, as well as health and safety professionals, should advise outdoor workers to wear white or light-colored clothes and hats/helmets. These pale colors increase the reflection of heat from the sun and can limit the heat strain experienced by the body. This multi-country series of field, clinical, and intervention studies raises another important issue; guidelines and policies should consider sun exposure as an important modifier of occupational and public health, not as one mere physical element to be included in the calculation of heat stress. Occupational and public health guidelines should be adapted based on exposure to solar radiation, fueled by a much-needed estimation of the associated burden of disease and injury.

## Figures and Tables

**Figure A4:**
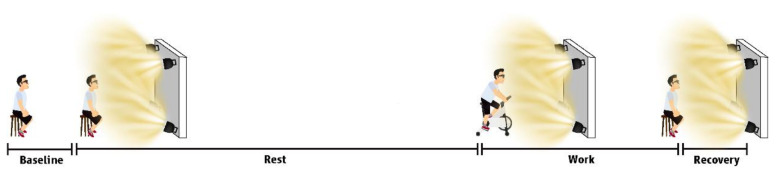
The experimental protocol of laboratory study.

## Appendix A

Study 1. Effects of solar radiation on the psychophysical stress experienced by workers who perform manual labor in construction and agriculture.

### Appendix A.1. Experimental Protocol

The experimental protocol (ClinicalTrials.gov (accessed on 1 July 2019) ID: NCT04160728) for these field experiments was approved by the Bioethical Committee of the School of Exercise Science of the University of Thessaly (protocol number: 1217) and the National Bioethical Review Board of Cyprus (protocol number: EEBK EP 2017.01.61) in accordance with the latest Declaration of Helsinki, except for registration in a database. The study involved monitoring 109 experienced (work experience: 4.8 ± 5.5 years) and acclimatized (living in the area for more than two months) agriculture and construction workers during four or five consecutive full work shifts. The construction work shifts monitored were (i) 00:00–11:00, (ii) 06:00–17:00, and (iii) 15:30–02:30, covering the entire day. In agriculture, the work shift monitored was 04:00–11:00. Prior to their participation in the study, written informed consent was obtained from all volunteers after a detailed explanation of all the procedures involved.

Self-reported age, body stature, and body mass were collected prior to the experiment. During the field study, continuous heart rate (HR), body core temperature (T_core_), and mean skin temperature (T_sk_) data were collected using wireless heart rate monitors (Polar Team2, Polar Electro Oy, Kempele, Finland), telemetric capsules (BodyCap, Caen, France), and wireless thermistors (iButtons type DS1921H, Maxim/Dallas Semiconductor Corp., Sunnyvale, CA, USA), respectively. Skin temperature data were collected from four body sites (chest, arm, thigh, and calf) and were expressed as a weighted average (T_sk_ = (0.3(chest + arm) + 0.2(thigh + calf))) [37]. Furthermore, continuous environmental data (air temperature (°C), globe temperature (°C), relative humidity (%), and air velocity (m/s)) were collected using portable weather stations (Kestrel 5400FW, Nielsen-Kellerman, Boothwyn, PA, USA) installed in close proximity to the workers. Thereafter, these environmental data were utilized to compute solar radiation (using air temperature, relative humidity, globe temperature, and air velocity) by a very well-known iterative method [38].

Real-time task analysis was utilized to evaluate labor intensity. This is a method based on the well-accepted time-motion analysis used to evaluate workers’ labor effort [13,39]. The main difference between the two methods is that real-time task analysis is performed live at the work site, while time-motion analysis is based on video recordings taken at the work site and analyzed at a later time. Work intensity (i.e., rest, low/medium/high-intensity) was based on the International Standard 8996 for the determination of metabolic rate [40]. Specifically, rest (65 W/m^2^) was characterized as any activity involving resting and/or sitting at ease. Low-intensity labor (100 W/m^2^) included activities incorporating “hand and arm work” or “hand and leg work” such as driving vehicles in normal conditions, machining, and casual walking (at a speed up to 2.5 km/h). Moderate-intensity labor (165 W/m^2^) included activities involving “hand and arm work” or “arm and leg work” or “arm and trunk work” such as working with construction equipment, weeding, picking fruits, or walking at a speed between 2.5 km/h to 5.5 km/h. High-intensity labor (230 W/m^2^) included any activity involving intense arm and trunk work, carrying heavy material, pushing or pulling heavily, or walking at a speed ranging between 5.5 km/h and 7 km/h.

The raw data collected were used to calculate hourly mean values. Thereafter, these averages were used to isolate work hours characterized by the same thermal stress but different solar radiation levels. Specifically, we isolated work hours characterized by 30 °C wet-bulb globe temperature (WBGT) rounded at 0.5 °C. At the end of each work shift, self-reported information regarding the psychophysical strain experienced throughout the work shift was collected using the Heat Strain Score Index [16]. A score beyond 18 in this tool indicates that a worker has a dangerously high risk of experiencing excessive heat strain [16].

We chose to use WBGT to describe thermal stress because it is the most widely used thermal index (10,900 times referred to or used according to Google Scholar metrics (15 October 2019)). Furthermore, it has been specifically designed for work activity assessments and adopted by reputable organizations in occupational safety and health worldwide, including the International Labour Organization [3]. It also incorporates all four environmental parameters (air temperature, relative humidity, wind speed, and solar radiation) for assessing the thermal stress experienced by workers who work in agriculture and construction.

### Appendix A.2. Perceived Thermal Radiation Scale

Alongside the aforementioned questionnaires, perceived thermal radiation was assessed using the following scale:

0. Non-detectable
1. Detectable
2. Very low
3. Low
4. Somewhat low
5. Neither low nor high
6. Somewhat high
7. High
8. Very high
9. Almost extreme
10. Extreme

### Appendix A.3. Statistical Analysis

Pearson’s correlation coefficient (r) was used to examine the relationships between solar radiation, physiological strain (HR, T_core_, and T_sk_) and labor intensity collected during the study. Similarly, Pearson’s correlation coefficient was used to investigate the relationships between perceived thermal radiation scale and subjective psychophysical parameters derived from the Heat Strain Score Index tool. A Bonferroni adjusted alpha of 0.003 was set for detecting statistically significant associations for this set of correlation analyses. Linear regression analyses with prediction intervals were used to examine if solar radiation levels can predict the physiological strain (HR, T_core_, and T_sk_) and labor intensity. Thereafter, solar radiation was categorized into three groups (indoor (0 to 160 W/m^2^), mixed (161 to 320 W/m^2^), and outdoor (>320 W/m^2^) environments) based on previous studies [41,42] reporting that 93–97% of the indoor environments are characterized by solar radiation levels ranging between 0 and 160 W/m^2^. We used solar radiation ranging between 161 and 320 W/m^2^ (twice the indoor environment) to define mixed environments (workers spending time both indoor and outdoor). Finally, solar radiation levels higher than 320 W/m^2^ were defined as outdoor environments. Thereafter, one-way ANOVA with post hoc (LSD) analysis was used to detect potential differences in HR, T_core_, T_sk_, and labor intensity between the three solar radiation categories (indoor, mixed, and outdoor environments).

Relative risks were calculated to investigate the risk of experiencing heat-related symptoms in indoor (0–160 W/m^2^) and outdoor (>160 W/m^2^) conditions. We considered T_sk_ values greater than 36 °C as indicative of high heat strain. This was based on previous studies reporting that the odds of progressive symptoms of heat strain increase rapidly after skin temperature reaches 36 °C [14,15], leading to a decreased capacity for physical work [43,44,45]. Thereafter, relative risks were calculated to investigate the probability that a worker has a dangerously high risk of experiencing high heat strain (based on a Heat Strain Score Index greater than 18 or T_sk_ above 36 °C) in indoor and outdoor conditions. The relative risks, their standard error, and 95% confidence interval were calculated based on a method described by Altman [46]. According to standard methodology [47], nil values were converted to 0.5 to avoid problems with the computation of effects or standard errors. The statistical significances of the identified relative risks values were determined by their accompanied 95% confidence interval [48].

Statistical analyses were conducted using both the SPSS v25.0 (IBM, Armonk, NY, USA) and Excel spreadsheets (Microsoft Office, Microsoft Corp., Redmond, WA, USA). The level of significance for these analyses was set at *p* < 0.05 unless otherwise specified.

### Appendix A.4. Results

**Table A1:** Anthropometric characteristics in Study 1.

	Mean	SD	Minimum	Maximum
Age (years)	33.6	8.2	18.0	56.0
Body mass (kg)	65.6	8.4	45.9	90.0
Body stature (m)	1.7	0.1	1.5	1.8
Body mass index (kg/m^2^)	23.7	3.0	17.3	33.4

**Table A2:** Association between “the perceived thermal radiation” and subjective psychophysical parameters derived from the Heat Strain Score Index. * indicates statistically significant association at a Bonferroni adjusted alpha of 0.003.

No.	Item	r	*p*
1	Heat Strain Score Index	0.517	<0.001 *
2	How would you describe the surface temperature of the surrounding equipment on your job site today?	0.430	<0.001 *
3	How much do you feel you sweat today while working?	0.340	<0.001 *
4	How fatigued do you feel during today’s work shift?	0.468	<0.001 *
5	How thirsty do you feel during today’s work shift?	0.376	<0.001 *
6	How much did the heat affect your ability to perform your job today?	0.356	<0.001 *

**Table A3:** Differences in psychophysical parameters between indoor and outdoor workers.

Symptom	Relative Risk	−95% CI	+95% CI
Heat Strain Score Index (danger level)	3.61	2.12	6.17
Mean skin temperature above 36 °C	10.16	4.25	24.33
Dizziness	4.44	0.61	1.34
Weakness	3.17	1.76	5.71
Any heat strain symptom	2.40	1.78	3.24

## Appendix B

Study 2. Effects of solar radiation on physiological responses and cognitive function at rest and during physical work.

### Appendix B.1. Experimental Protocol

This was a single-blind randomized controlled trial (ClinicalTrials.gov (accessed on 1 July 2019) ID: NCT04160741). The experimental protocol was approved by the Bioethical Committee of the School of Exercise Science of the University of Thessaly (protocol number: 1303) in accordance with the Declaration of Helsinki. Seven healthy non-smoking males (see “Sample Size Calculation”) participated in the experiments. Prior to their participation in the study, written informed consent was obtained from all volunteers after a detailed explanation of all the procedures involved.

Volunteers visited the laboratory on four consecutive days and were exposed to four different environmental conditions (Table A4) inside an environmental chamber. Thermal stress was set at 30 °C WBGT for hot and 20 °C WBGT for temperate conditions [49]. The environmental chamber used (2.85 m × 2.85 m × 4 m) was accurate to ±0.5 °C, ±3% relative humidity, ±10 W/m^2^ solar radiation, and ±0.1 m/sec wind speed within the tested range. A WBGT meter (Kestrel 5400FW, Nielsen-Kellerman, Boothwyn, PA, USA) was utilized to confirm the reliability of the simulated environment at head, chest, and feet levels. Solar radiation was measured with a solar power meter (TES 1333R, TES, Taipei, Taiwan) at head, chest, and feet level.

**Table A4:** Simulated environmental conditions during lab experiments.

Environment	Thermal Stress	AT (°C)	RH (%)	WS (m/s)	SR (W/m^2^)	WBGT (°C)
Outdoor	Hot	32.6	30	0.5	800	30
	Temperate	19.1	30	0.5	800	20
Indoor	Hot	40.6	30	0.5	0	30
	Temperate	28.5	30	0.5	0	20

AT = air temperature; RH = relative humidity; WS = wind speed; SR = solar radiation; WBGT = wet-bulb globe temperature.

Each volunteer underwent the aforementioned four environmental scenarios in random order. To minimize participant bias in the cognitive performance and the subjective assessments used (see below), the true purpose of the study was hidden from the volunteers. Instead, they were told that the study investigated the impact of heat (not specifically solar radiation) on human performance. Once the data collection was completed, all volunteers were informed about the true purpose of the study and gave their permission to analyze and publish these data.

Each of the aforementioned four trials included a baseline assessment of cognitive performance (outside the environmental chamber; duration: approx. 20 min) and three hours and twenty minutes of data collection inside the environmental chamber with three main time periods: rest (two hours), work (one hour), and recovery (twenty minutes) (Figure A4). Volunteers entered the environmental chamber five minutes prior to the start of data collection to apply and set up the equipment and sensors on their bodies.

All trials took place during the same time of the day for each participant, following an 8-h fast. Participants were requested to arrive at the laboratory in a euhydrated state and to avoid caffeine and alcohol consumption for at least twelve hours before the experiments, as well as to avoid salt and sugar consumption eight hours before the experiments. Based on existing guidelines [50], euhydration was defined as urine specific gravity < 1.020 (urine sample taken at participants’ arrival), which was assessed using a handheld refractometer (ATAGO Ltd., Tokyo, Japan). Water consumption was prohibited during the experimental protocol. The same clothing consisting of a light-blue t-shirt (100% cotton), a black exercise pair of shorts (100% polyester), and a pair of medium-high socks (100% cotton) were used by volunteers for all experiments (estimated clothing insulation = 0.38 clo (shoes = 0.04 clo; socks = 0.04 clo; underwear = 0.04 clo; t-shirt = 0.18 clo; shorts = 0.08 clo)) [51]. Additionally, a pair of sunglasses was worn throughout the experiments.

Two days prior to the experiments, we assessed body stature (Seca 213; seca GmbH & Co. KG; Hamburg, Germany), body mass (BC1000, Tanita corporation, Tokyo, Japan), as well as body fat and lean mass (Dual energy X-ray absorptiometry (DEXA); Lunar DPX Madison, GE Healthcare, Madison, WI, USA). During the study, continuous HR, T_core_, T_sk_, local skin blood flow, and local sweat rate were measured. Specifically, HR was collected using wireless heart rate monitors (Polar Team2, Polar Electro Oy, Kempele, Finland). T_core_ was recorded using telemetric capsules (BodyCap, Caen, France). Skin temperature from four sites was measured using wireless thermistors (iButtons type DS1921H, Maxim/Dallas Semiconductor Corp., Sunnyvale, CA, USA) and was expressed as T_sk_ according to Ramanathan (T_sk_ = 0.3(chest + arm) + 0.2(thigh + leg)) [37]. Skin blood flow was measured with a laser Doppler flowmeter (PeriFlux 4000, Perimed, Stockholm, Sweden) at the right forearm (brachioradialis) and leg (gastrocnemius), ensuring no direct exposure to solar radiation. The probe (PROBE 413 Integrating Probe, Perimed, Stockholm, Sweden) was held in place with a plastic holder (PH 13, Perimed, Stockholm, Sweden). Sweat rate was measured at three regions (forehead (subject to direct solar radiation), thigh/quadricep (subject to indirect solar radiation) [52], and arm/bicep (subject to indirect solar radiation)) using the ventilated capsule method. Thermal comfort (1 = comfortable; 5 = extremely uncomfortable), thermal sensation (−3 = cold; +3 = hot), and perceived exertion (6 = no exertion at all; 20 = maximal exertion) [53], alongside cognitive performance (see “Appendix B.3. Assessment of Cognitive Performance”) were assessed at baseline, before work (at 01:40:00), and following work (at 03:00:00).

### Appendix B.2. Sample Size Calculation

The minimum required sample size for investigating “differences between dependent means” was calculated using the difference in T_sk_ (see results of study 1) between workers working in indoor (34.6 ± 0.9 °C) and outdoor (35.8 ± 1.0 °C) conditions. Using these data, an effect size (dz) of 1.27 for the differences between indoor and outdoor conditions was computed. Assuming an α of 0.05 and β of 0.90, seven participants would provide enough power to detect a statistical difference of a similar magnitude (G*Power Version 3.1.9.2) [54]. Based on these calculations, a total of seven healthy individuals volunteered and were recruited for this study.

### Appendix B.3. Assessment of Cognitive Performance

Familiarization for all tests was undertaken one week prior to the experiments. During these familiarizations, as well as during the data collection, volunteers were isolated in a room with no external visual and/or acoustic stimuli. Screen brightness and sound volume remained constant throughout the experiments. The same sequence of cognitive performance tests (1st: reaction time in acoustic stimuli; 2nd: reaction time in visual stimuli; 3rd: memory test; 4th: divided attention; and 5th: vigilance) was followed throughout the experiments. Approximately 20 min were required to complete all the cognitive tests. These tests were repeated three times: at baseline (20 min before entering the chamber), before work (at 01:40:00), and following work (at 03:00:00).

- Vigilance Test: We used a well-known vigilance test described in the Test for Attentional Performance [55] that has been well-accepted in the literature [56]. To run the test, we developed a computer software (freely available at www.famelab.gr/research/downloads/ accessed on 1 July 2019). In brief, this task involves two squares arranged vertically. A pattern jumps from one square to the other. Sometimes the pattern repeats in the same square. When this happens, volunteers are instructed to touch the screen of a tablet computer as fast as possible. The total duration of this test was set to six minutes.
- Divided Attention: We used a well-known vigilance test described in the Test for Attentional Performance [55] that has been well-accepted in the literature [57]. To run the test, we developed a computer software (freely available at www.famelab.gr/research/downloads/ accessed on 1 July 2019). In brief, this task involves both auditory and visual stimuli in parallel. During the test, a number of visual stimuli (crosses) appear in a random configuration in a 4 × 4 matrix. At the same time, volunteers hear high- and low-pitch beeps in a random order. The aim is to touch the screen of a tablet computer as fast as possible when crosses form a square and, at the same time, two high- or low-pitch beeps are emitted twice in a row.
- Memory Test: We developed a computer software (freely available at www.famelab.gr/research/downloads/ accessed on 1 July 2019) to perform Sternberg’s Memory Test [58]. In this well-known test, volunteers had to observe carefully a random sequence of one to six digits (ranging from 0 to 9) in white font and displayed for 1.2 s each. Following a 2 s delay, a random digit (from the numbers that were previously presented) in yellow font was presented. Participants had to indicate whether the yellow digit was part of the sequence of numbers presented or not by pressing the “yes” or “no” buttons on the screen of a tablet computer as fast as possible. Each test included a total of 24 trials.
- Reaction Time: We developed a computer software (freely available at www.famelab.gr/research/downloads/ accessed on 1 July 2019) to assess reaction time. Participants were requested to place their finger on the screen of a tablet computer and to remove it as fast as possible after receiving a visual (i.e., screen turning from black to yellow) or acoustic (i.e., a loud beep was heard) stimulus.

### Appendix B.4. Work Intensity

Volunteers were instructed to cycle at 100 W (52.5 ± 3.8 W/m^2^) for 60 min. A cycle ergometer (CycleOps 400 Pro Serie Indoor Cycle, Fitchburg, MA, USA) combined with a commercially available software Rouvy (VirtualTraining, Vimperk, Czech Republic) was used to ensure constant work intensity throughout the experiments. We adopted an absolute work intensity in W (i.e., not expressed as a function of body surface area) because that is more representative for workers who perform manual labor in occupational settings (i.e., workers with different anthropometric characteristics are expected to produce the same work output).

The metabolic rate during work was approximately 300 W/m^2^ (297 ± 14.1 W/m^2^), corresponding to a broad spectrum of moderate-intensity manual labor tasks, including “coal mining, drilling coal and/or rock” (308.2 W/m^2^), “farming, moderate effort” (279.12 W/m^2^), “fishing, commercial, moderate effort” (290.75 W/m^2^), “forestry, ax chopping, slow” (290.75 W/m^2^), “machine tooling, operating punch press, moderate effort” (290.75 W/m^2^), “shoveling, less than 10 lbs/min, moderate effort” (290.75 W/m^2^), “steel mill, moderate effort” (308.2 W/m^2^), and “walking, carrying objects about 25 to 49 lbs” (290.75 W/m^2^) [51,59,60,61]. The metabolic rate (in W/m^2^) was calculated by applying body surface area [62] in an iterative method to the well-known equation developed by Fiala [51,63].

### Appendix B.5. Solar Radiation

The amount of radiant heat absorbed by the human body during typical outdoor work varies considerably based on body posture [64]. A previous study identified that 81.4% of the work shift time is spent crouching, 12% standing, and 6.6% sitting [51]. Based on this information, the participants were placed in a crouching position throughout the data collection: sitting on a high stool during rest and recovery, and cycling during work. The solar radiation level was set at 800 W/m^2^, which is a typical level of solar radiation during work under clear sky conditions [13]. Solar radiation was simulated by four Compact Source Iodide lamps previously tested and used for this purpose [65]. These lamps are characterized by high light efficacy (>90 lm/W) and good balance in spectral qualities (the mean difference between solar radiation and these lamps was found to be just 0.01% across six spectral bands), and thus they are capable of simulating sun light [65]. Two lamps were located at the height of 2.2 m, facing participants at about 50° angle (more representative for the average sun angle). The other two lamps were located 50 cm above the ground to ensure equal distribution of radiation waves on the participants’ bodies. Interindividual differences in body stature between participants were addressed by personalized adjustments in the angle and intensity (using a potentiometer) of the lamps.

### Appendix B.6. Statistical Analysis

Effect sizes were calculated to investigate the differences in cognitive performance and physiological parameters between outdoor hot and indoor hot, as well as between outdoor temperate and indoor temperate environments (Table A5). The magnitude of effect sizes was determined as follows: d (0.01) = very small; d (0.2) = small; d (0.5) = medium; d (0.8) = large; d (1.2) = very large; and d (2.0) = huge [66]. Pearson’s correlation coefficient r was used to examine the relationships between mean skin temperature, core temperature, and cognitive performance. A Bonferroni adjusted alpha of 0.006 was set for detecting statistically significant associations for this set of correlation analyses. Statistical analyses were conducted using both the SPSS v25.0 (IBM, Armonk, NY, USA) and Excel spreadsheets (Microsoft Office, Microsoft Corp., Redmond, WA, USA). The level of significance for these analyses was set at *p* < 0.05 unless otherwise specified.

### Appendix B.7. Results

**Table A5:** The effect of solar radiation on physiological responses and cognitive performance.

		Entire Protocol		Entire Rest		Entire Work		Effect Size (d)		
		Mean	SD	Mean	SD	Mean	SD	Entire	Rest	Work
**Core Temperature** (°C):										
	Outdoor–hot	37.7	0.7	37.2	0.3	38.3	0.6	0.06	0.01	0.25
	Indoor–hot	37.6	0.6	37.2	0.3	38.1	0.5			
	Outdoor–temperate	37.2	0.4	37.0	0.3	37.6	0.3	0.02	0.17	0.01
	Indoor-temperate	37.2	0.5	36.9	0.2	37.6	0.5			
**Mean Skin Temperature** (°C):										
	Outdoor–hot	38.2	0.8	38.2	0.8	38.5	0.5	1.57	2.12	1.64
	Indoor–hot	36.6	1.0	36.6	0.7	36.8	1.2			
	Outdoor–temperate	35.0	1.2	35.2	1.2	34.5	1.1	0.44	0.64	0.16
	Indoor-temperate	34.4	1.3	34.4	1.1	34.3	1.5			
**Heart Rate (beats/min):**										
	Outdoor–hot	114.0	34.2	90.5	5.6	160.2	21.2	0.13	0.75	0.14
	Indoor–hot	109.1	34.6	85.1	7.6	157.2	18.6			
	Outdoor–temperate	102.6	29.0	80.8	4.6	138.9	16.9	0.22	1.59	0.21
	Indoor-temperate	95.4	31.8	71.3	6.3	135.1	17.2			
**Skin Blood Flow (arbitrary perfusion units, A(PU))** ***Arm:***										
	Outdoor–hot	121.9	182.5	32.4	32.6	320.0	229.5	0.05	0.62	0.04
	Indoor–hot	130.7	142.7	53.3	30.3	312.1	148.3			
	Outdoor–temperate	67.3	101.9	10.3	15.7	202.0	95.4	0.24	0.16	0.75
	Indoor-temperate	103.2	166.8	14.4	30.2	314.2	172.1			
***Leg***:										
	Outdoor–hot	104.1	52.4	78.7	31.0	157.8	55.0	0.51	0.81	0.70
	Indoor–hot	78.9	39.6	55.4	21.4	124.5	30.1			
	Outdoor–temperate	69.0	51.3	45.6	33.3	126.1	43.3	0.28	0.54	0.35
	Indoor-temperate	54.0	46.9	29.4	21.5	109.5	44.1			
**Sweat Rate (mg/cm^2^/min)** ***Forehead*:**										
	Outdoor–hot	2.6	1.2	2.4	1.0	2.8	1.3	0.49	0.65	0.21
	Indoor–hot	2.1	0.6	1.8	0.5	2.6	0.6			
	Outdoor–temperate	1.1	0.7	1.0	0.8	1.3	0.7	0.33	0.14	0.61
	Indoor–temperate	1.4	0.9	1.2	0.9	1.8	0.9			
***Arm:***										
	Outdoor–hot	1.3	0.6	1.2	0.5	1.4	0.6	0.10	0.20	0.09
	Indoor–hot	1.2	0.6	1.1	0.6	1.5	0.6			
	Outdoor–temperate	0.6	0.3	0.4	0.3	0.9	0.3	0.35	0.39	0.63
	Indoor-temperate	0.5	0.2	0.3	0.1	0.7	0.2			
***Thigh:***										
	Outdoor–hot	1.1	0.5	1.0	0.4	1.3	0.7	0.56	0.68	0.58
	Indoor–hot	0.8	0.3	0.7	0.3	0.9	0.4			
	Outdoor–temperate	0.5	0.5	0.3	0.1	1.0	0.7	0.29	0.70	0.52
	Indoor-temperate	0.3	0.3	0.2	0.1	0.6	0.4			
**Divided Attention (% Mistakes):**										
	Outdoor–hot	30.4	21.0	29.0	26.6	31.8	15.5	0.47	0.49	0.45
	Indoor–hot	21.0	15.9	17.4	15.4	24.0	16.8			
	Outdoor–temperate	17.9	15.6	15.1	18.1	20.7	13.5	0.12	0.02	0.21
	Indoor-temperate	16.1	13.2	14.7	12.4	17.5	14.9			
**Vigilance (% Mistakes):**										
	Outdoor–hot	14.6	12.7	13.5	11.6	15.9	14.8	0.48	1.18	0.14
	Indoor–hot	8.7	10.1	2.7	3.2	13.9	11.2			
	Outdoor–temperate	10.8	15.7	9.7	15.0	11.8	17.6	0.44	0.35	0.50
	Indoor-temperate	5.1	6.0	5.2	7.2	4.9	5.0			
**Memory test (% Mistakes):**										
	Outdoor–hot	8.7	6.2	8.3	7.6	9.0	4.9	0.21	0.56	0.12
	Indoor–hot	7.1	8.1	4.2	5.9	10.0	9.6			
	Outdoor–temperate	4.5	5.0	3.0	4.6	6.0	5.3	0.09	0.40	0.45
	Indoor-temperate	5.0	5.5	5.8	8.1	4.2	0.0			
**Auditory Reaction Time (ms):**										
	Outdoor–hot	527.0	71.0	517.9	80.0	536.1	65.7	0.35	0.30	0.44
	Indoor–hot	501.2	64.0	491.3	85.4	511.1	37.0			
	Outdoor–temperate	474.5	54.5	475.6	61.5	473.4	51.4	0.04	0.16	0.04
	Indoor-temperate	477.6	93.8	486.3	63.5	468.9	121.8			
**Visual Reaction Time (ms):**										
	Outdoor–hot	214.7	25.7	216.6	26.5	212.9	26.8	0.12	0.32	0.05
	Indoor–hot	211.1	28.7	207.7	25.1	214.4	33.5			
	Outdoor–temperate	218.6	26.5	227.4	32.1	209.7	17.8	0.27	0.11	0.44
	Indoor-temperate	236.7	84.9	223.3	34.6	250.1	118.4			
**Perceived Exertion (6 = none; 20 = maximum):**										
	Outdoor–hot	11.9	4.3	9.6	2.6	16.9	2.3	0.09	0.08	0.15
	Indoor–hot	11.5	4.5	9.3	3.1	16.5	2.7			
	Outdoor–temperate	9.5	2.6	8.1	1.5	12.3	2.2	0.22	0.46	0.23
	Indoor-temperate	8.9	2.5	7.4	1.3	11.8	1.8			
**Thermal Sensation (−3 = very cold; +3 = very hot):**										
	Outdoor–hot	2.5	0.6	2.4	0.6	2.8	0.4	0.00	0.08	0.18
	Indoor–hot	2.5	0.5	2.4	0.5	2.8	0.5			
	Outdoor–temperate	1.1	0.8	0.9	0.6	1.6	0.9	0.70	1.09	0.52
	Indoor-temperate	0.6	0.7	0.3	0.5	1.2	0.6			
**Thermal Comfort (1 = comfortable; 5 = very uncomfortable):**										
	Outdoor–hot	3.2	1.0	2.9	0.9	4.0	0.9	0.20	0.26	0.25
	Indoor–hot	3.4	0.8	3.1	0.7	4.2	0.7			
	Outdoor–temperate	2.2	0.8	1.9	0.5	2.8	0.7	0.52	0.79	0.48
	Indoor-temperate	1.8	0.8	1.4	0.5	2.4	0.8			
**Notes:**										
	Positive values correspond to an incremental effect of solar radiation on this parameter.									
	Negative values correspond to a diminishing effect of the solar radiation on this parameter.									
	Effect size values were grouped to the closest category.									
**Effect Size (d):**										
	**Very Small**	**Small**	**Medium**	**Large**	**Very Large**	**Huge**				
positive	0.01	0.2	0.5	0.8	1.2	2.0				
negative	0.01	0.2	0.5	0.8	1.2	2.0				

**Table A6:** Associations between cognitive performance and either the mean skin temperature or core temperatures. * indicates statistically significant association at a Bonferroni adjusted alpha of 0.006.

Parameter	Mean Skin Temperature		Core Temperature	
	r	*p*	r	*p*
Vigilance (more mistakes)	0.137	0.229	0.297	0.008
Divided Attention (more mistakes)	0.248	0.027	0.274	0.015
Memory (more mistakes)	0.373	0.002	0.335	0.006 *
Perceived Exertion (worse)	0.333	0.003	0.804	<0.001 *
Thermal Sensation (worse)	0.562	<0.001	0.523	<0.001 *
Thermal Comfort (worse)	0.467	<0.001	0.671	<0.001 *
Reaction time in auditory stimuli (slower)	0.377	0.001	0.364	0.001 *
Reaction time in visual stimuli	0.18	0.873	−0.025	0.873

## Appendix C

Study 3. Identifying factors increasing the adverse effects of sun exposure experienced by agriculture and construction workers.

### Appendix C.1. Experimental Protocol

The experimental protocol for these field experiments was approved by the National Bioethical Review Board of Cyprus (protocol number: EEBK EP 2017.01.61) in accordance with the Declaration of Helsinki. An observational study was conducted in Cyprus to identify possible factors affecting the radiant heat exchange (i.e., between workers’ bodies and the surrounding environment) leading to increased heat strain in outdoor occupational settings. For this reason, 78 agriculture workers (112 full work shifts) from seven countries (Bangladesh, Cyprus, Egypt, India, Philippines, Romania, and Vietnam) were monitored over a period of three months. Specifically, video recordings (Hero 5 black, GoPro, San Mateo, CA, USA) were used to examine workers’ clothing during actual work shifts performed outdoors. Munsell color system (0 = black to 10 = white) was used to categorize workers’ clothing (dark color = 0 to 5 and light color = 6 to 10) [67]. To minimize examiner bias, an examination of workers’ clothing was conducted independently by two investigators. Any conflicts were resolved by discussion.

### Appendix C.2. Results

**Table A7:** Anthropometric characteristics of the participants in Study 4.

	Mean	SD	Minimum	Maximum
**Agriculture**				
Age (years)	39.2	11.8	21.0	56.0
Body mass (kg)	77.0	16.2	54.2	100.5
Body stature (m)	1.68	0.09	1.53	1.81
Body mass index (kg/m^2^)	27.1	5.2	20.9	36.5
**Construction**				
Age (years)	33.6	8.4	18.0	52.0
Body mass (kg)	65.4	8.6	50.0	90.0
Body stature (m)	1.66	0.06	1.54	1.80
Body mass index (kg/m^2^)	23.9	3.1	17.3	33.4

## Appendix D

Study 4. Interventions to mitigate the sunlight-induced heat strain experienced by workers who work in agriculture and construction.

### Appendix D.1. Experimental Protocol

The experimental protocol (ClinicalTrials.gov (accessed on 1 July 2019) ID: NCT04160728) for these field experiments was approved by the Bioethical Committee of the School of Exercise Science of the University of Thessaly (protocol number: 1217) and the National Bioethical Review Board of Cyprus (protocol number: EEBK EP 2017.01.61) in accordance with the Declaration of Helsinki. Two experimental field studies were conducted in Cyprus (agriculture workers) and Qatar (construction workers) to investigate whether providing workers with light-colored clothes is able to mitigate the sunlight-induced heat strain they experience during their work shifts. The idea behind this study was to find a feasible and economically viable mitigation strategy able to help workers in an occupational setting.

### Appendix D.2. Intervention in Agriculture

A group of six (see “Sample Size Calculation”) agriculture workers (performing tasks at ~200 W/m^2^) from Cyprus volunteered to participate in the study. The study involved monitoring two consecutive full work shifts (“business as usual” and “white clothing” scenarios). All testing procedures were similar between the two scenarios, with the only difference being that workers during the white clothing scenario were provided with white hats and t-shirts (all 100% cotton), as well as they were instructed to wear light-colored pants. One day prior to the start of data collection, volunteers underwent a familiarization session that included information regarding all data collection procedures. Written informed consent was obtained from all volunteers prior to their participation in the study.

### Appendix D.3. Intervention in Construction

A group of 41 (see “Sample Size Calculation”) construction workers (performing tasks at ~100 W/m^2^) from Qatar volunteered to participate in the study. The study involved monitoring two consecutive full work shifts (“business as usual” and “white coverall” scenarios). All testing procedures were similar between the two scenarios, with the only difference being that workers during the white coverall (half: 100% cotton; half: 65% cotton and 35% polyester) scenario were provided with white coveralls (estimated clothing insulation = 0.91 clo (shoes = 0.04 clo; socks = 0.04 clo; underwear = 0.04 clo; t-shirt = 0.18 clo; coverall = 0.61 clo)) [12,51]. One week prior to the start of data collection, volunteers underwent a familiarization session that included information regarding all data collection procedures. Written informed consent was obtained from all volunteers prior to their participation in the study.

### Appendix D.4. Data Collection

Anthropometric data (age; body stature (Seca 213; seca GmbH & Co. KG; Hamburg, Germany) and body mass (BC1000, Tanita Corporation, Tokyo, Japan)) were collected two days prior to the experiment. During the study, continuous heart rate, T_core_ and T_sk_ data were collected using wireless heart rate monitors (Polar Team2. Polar Electro Oy, Kempele, Finland), telemetric capsules (BodyCap, Caen, France), and wireless thermistors (iButtons type DS1921H, Maxim/Dallas Semiconductor Corp., Sunnyvale, CA, USA), respectively. Skin temperature data were collected from four sites (chest, arm, thigh, and lower leg) and were expressed as T_sk_ (T_sk_ = 0.3(chest + arm) + 0.2(thigh + leg)) [37]. Furthermore, continuous environmental data (WBGT and solar radiation) were collected using a portable weather station (Kestrel 5400FW, Nielsen-Kellerman, Boothwyn, PA, USA) and a handheld solar power meter (TES 1333R, TES, Taipei, Taiwan), respectively. Video recordings (Hero 5 black, GoPro, San Mateo, CA, USA) were used to calculate work intensity by means of time-motion analysis in agriculture [13], while real-time task analysis was utilized to evaluate labor intensity in construction.

### Appendix D.5. Sample Size Calculation

The minimum required sample size for investigating “differences between dependent means” was calculated using the difference in T_sk_ (see results of study 2; Table A1) between Outdoor-Heat (38.2 ± 0.8 °C) and Indoor-Heat (36.6 ± 1.0 °C) environmental scenarios. Using these data, an effect size (dz) of 1.88 for the differences between Outdoor-Heat and Indoor-Heat environmental scenarios was computed. Assuming an α of 0.05 and β of 0.95, five participants would provide enough power to detect a statistical difference of a similar magnitude (G*Power Version 3.1.9.2) [54]. Based on these calculations, a total of six healthy individuals volunteered and were recruited for the interventions conducted in agriculture, while 41 more individuals volunteered for the interventions in the construction sector.

### Appendix D.6. Statistical Analysis

Effect sizes were calculated to examine possible differences in the physiological heat strain experienced by the workers between “business as usual” and “white clothing” scenarios. The magnitude of effect sizes was determined as follows: d (0.01) = very small; d (0.2) = small; d (0.5) = medium; d (0.8) = large; d (1.2) = very large; and d (2.0) = huge [66]. Statistical analyses were conducted using Excel spreadsheets (Microsoft Office, Microsoft Corp., Redmond, WA, USA).

### Appendix D.7. Results

**Table A8:** Differences in workers’ physiological responses between “business as usual” and “white clothing” scenarios.

				Entire Protocol			Effect Size
				Mean	SD		d
**Agriculture**							
Mean Skin Temperature (°C):			Business as usual	33.6	1.6		0.23
			White clothing	33.2	1.7		
Heart Rate (beats/min):			Business as usual	96.1	13.9		0.23
			White clothing	92.9	10.6		
Perceived exertion (6 = no exertion; 20 = max exertion):			Business as usual	11.3	1.4		0.12
			White clothing	11.2	1.1		
Thermal Sensation (−3 = very cold; +3 = very hot):			Business as usual	1.4	0.2		0.26
			White clothing	1.3	0.4		
Thermal Comfort (1 = comfortable; 5 = very uncomfortable):			Business as usual	2.3	0.5		0.00
			White clothing	2.3	0.5		
**Construction**							
Mean Skin Temperature (°C):			Business as usual	35.4	0.6		0.34
			White clothing	35.2	0.7		
Heart Rate (beats/min):			Business as usual	91.0	7.8		0.04
			White clothing	90.7	9.3		
**Notes:**							
	Positive values correspond to an incremental effect of intervention on this parameter.						
	Negative values correspond to a diminishing effect of the intervention on this parameter.						
	Effect size values were grouped to the closest category.						
**Effect Size (d)**							
	**Very Small**	**Small**	**Medium**	**Large**	**Very Large**	**Huge**	
positive	0.01	0.2	0.5	0.8	1.2	2.0	
negative	0.01	0.2	0.5	0.8	1.2	2.0	
